# The secrets of menstrual blood: emerging frontiers from diagnostic tools to stem cell therapies

**DOI:** 10.3389/fcell.2025.1623959

**Published:** 2025-08-20

**Authors:** Yige Feng, Yujie He

**Affiliations:** ^1^ The First Clinical College, Shanxi Medical University, Taiyuan, Shanxi, China; ^2^ The Reproductive Medicine Center, The First Hospital of Shanxi Medical University, Taiyuan, Shanxi, China

**Keywords:** menstruation, diagnosis, therapy, stem cells, research progress

## Abstract

Menstrual blood (MB), a biofluid rich in diverse cell types and biomolecules, has emerged as a vital resource for investigating female reproductive health and diseases because of its unique composition and noninvasive accessibility. This review explores the potential of MB in medical research and clinical applications, focusing on its diagnostic and therapeutic prospects. For disease diagnosis, MB offers a noninvasive sampling method for identifying biomarkers in endometriosis, cervical cancer, and other gynecological conditions. Therapeutically, stem cells derived from MB (menstrual blood-derived stem cells, MenSCs) exhibit pluripotency, high proliferative capacity, and low immunogenicity, positioning them as promising candidates in regenerative medicine. Preclinical and clinical studies have demonstrated the efficacy of MenSCs in treating infertility, premature ovarian insufficiency, intrauterine adhesions, hepatic disorders, cutaneous injuries, and neurological diseases. MenSCs also exert therapeutic effects through paracrine mechanisms by releasing cytokines and exosomes that modulate immunity, attenuate inflammation, and promote tissue repair. Despite existing challenges, MenSCs hold substantial promise for developing novel therapeutic strategies across multiple disease domains.

## 1 Background

Menstruation is a central physiological phenomenon of the female reproductive system characterized by cyclic shedding and bleeding of the endometrium triggered by ovarian cyclical changes. Throughout the reproductive lifespan, women typically undergo over 400 cycles of endometrial regeneration, differentiation, and shedding (Lv et al., 2018). These cyclic processes are regulated primarily by the hypothalamus‒pituitary‒ovary axis.

Menstrual blood (MB) is a complex biofluid containing blood from endometrial spiral arteries, vaginal secretions, and endometrial cells (van der Molen et al., 2014; Yang et al., 2012). Notably, MB results in significant differences in the concentrations of specific biomolecules and cellular compositions compared with those of peripheral blood (PB) (Iribarne-Durán et al., 2020; Hosseini et al., 2019). The shed endometrial cells encompass diverse types, including stromal cells, epithelial cells, vascular cells, and immune cells. These components not only reflect endometrial homeostasis but also provide critical insights for investigating reproductive health and disease mechanisms.

Given the heterogeneous and multifaceted composition of MB, research interest in this field has expanded substantially in recent years. This review systematically examines the applications of MB over the past decade in disease diagnostics, therapeutic development, pathogenesis elucidation, and genetic research. Furthermore, it evaluates the current challenges in clinical translation and outlines future directions to advance MB-based biomedical innovations.

## 2 Disease diagnosis

As a diagnostic specimen, MB offers multiple advantages, including ease of sampling, noninvasiveness, self-collection capability, periodic availability, and fewer ethical concerns (Hosoya et al., 2023). In terms of population acceptability, Wong et al. evaluated the willingness of 5,000 women to use MB as a diagnostic sample, with the results indicating that 87% of participants supported its utilization for testing (Wong et al., 2018). Another study by Budukh further validated the feasibility of MB as a screening specimen for cervical cancer (Budukh et al., 2018). This noninvasive collection method not only eliminates the discomfort associated with traditional sampling procedures but also empowers women to flexibly manage their daily activities. Consequently, MB has significant advantages as a diagnostic specimen applicable to all menstruating women.

### 2.1 Cervical cancer

Cervical cancer remains a leading cause of cancer-related mortality among women globally, accounting for approximately 25% of all female malignancies (Harro et al., 2001). With a mortality rate second only to that of breast cancer, it is a critical public health concern (Jin et al., 1999). Human papillomavirus (HPV) is the primary etiological agent, driving lesion progression from low-grade cervical intraepithelial neoplasia (CIN1) to high-grade neoplasia (CIN2/3) and microinvasive lesions, ultimately leading to invasive cervical cancer (Holowaty et al., 1999). Understanding this pathophysiological trajectory is essential for developing diagnostic, therapeutic, and preventive strategies. Early detection and intervention during the precancerous phase are pivotal to halting disease progression (Spitzer, 1998).

Current screening and diagnostic methods for cervical cancer rely primarily on cytology and histology. From the conventional Papanicolaou smear to improved liquid-based cytology and automated processes, these approaches have significantly reduced cervical cancer mortality in developed countries. However, owing to the high false-positive rates of cytology, colposcopy with directed biopsy is often required for further evaluation. While colposcopy can detect low- and high-grade dysplasia, its sensitivity for identifying microinvasive lesions remains limited. In cases with inconclusive findings or incomplete visualization of the squamocolumnar junction, cone biopsy is necessary to confirm diagnoses through histopathological identification of HPV-associated features. Additionally, molecular detection of high-risk HPV DNA sequences has been introduced in recent years, enhancing diagnostic sensitivity and specificity. Emerging HPV testing technologies, such as next-generation sequencing (NGS), enable hypothesis-free comprehensive genetic analysis, offering novel strategies for cervical cancer screening.

Currently, cervical cancer screening relies on cytology and histology. These methods, ranging from conventional Pap smears to advanced liquid-based cytology and automated systems, have significantly reduced mortality in developed nations (Hu and Ma, 2018). However, the high false-positive rates of cytology necessitate confirmatory colposcopy with directed biopsy. While colposcopy identifies dysplasia, its sensitivity for detecting microinvasive lesions is limited, particularly in cases with incomplete visualization of the squamocolumnar junction, which requires cone biopsy for histopathological confirmation (Burd, 2003). Molecular HPV DNA testing has emerged as a complementary tool, enhancing diagnostic accuracy (Burd, 2003). Next-generation sequencing (NGS) represents a novel frontier, enabling comprehensive genetic analysis without prior hypotheses (Tsang et al., 2022; Pei et al., 2023).

Despite these advancements, all current methods require invasive sampling, which can cause patient discomfort and psychological stress. Emerging studies on HPV detection in MB propose a noninvasive alternative. Zhang et al. (2021) collected MB via sanitary pads from premenopausal women with high-risk HPV positivity, extracted DNA, and performed next-generation sequencing (NGS)-based HPV genotyping. Compared with conventional cervical smears, MB-based testing achieved 97.7% sensitivity, identifying additional HPV genotypes, multiple infections, and true-negative cases. It also accurately detected high-risk HPV in routine false-negative test results.

Similarly, Tsang et al. (2024) used NGS to analyze MB samples from CIN/HPV-positive patients and reported a 66.7% sensitivity for high-risk HPV detection. Wong et al. (2018) further explored HPV DNA and genetic polymorphisms in MB from CIN/HPV patients and healthy controls. They reported that 83% of the participants tested HPV-positive and successfully genotyped, whereas 4% of the controls tested HPV-positive. Notably, TAP1 gene polymorphisms (I333V and D637G) in MB were linked to a reduced risk of high-grade intraepithelial neoplasia, offering actionable insights for clinical management and resource allocation.

### 2.2 Endometriosis

Endometriosis is a chronic disease characterized by the ectopic growth of endometrial-like tissue outside the uterine cavity, resulting in inflammatory, hormone dependent, immunological, systemic, and heterogeneous pathophysiological features, predominantly affecting reproductive-aged women (Sinaii et al., 2008). Its primary symptoms include pelvic pain, which may manifest as dysmenorrhea, dyspareunia, or chronic pelvic pain, often accompanied by overlapping symptoms (e.g., urinary or gastrointestinal manifestations), complicating clinical diagnosis (Parasar et al., 2017). Owing to symptom overlap with other gynecological conditions (e.g., ovarian cysts, uterine fibroids, or pelvic inflammatory disease sequelae) or chronic pain syndromes, a definitive diagnosis requires the integration of patient history, clinical examination, and imaging (Sinaii et al., 2008; Ballweg, 2004). However, the gold-standard diagnostic method—laparoscopy—is limited by its invasiveness (Parasar et al., 2017). Current research on noninvasive biomarker-based diagnostic approaches remains underdeveloped (Falcone and Flyckt, 2018).

The discovery of aromatase has opened new avenues for noninvasive endometriosis diagnosis. Noble et al. (1996) demonstrated aromatase expression in both the eutopic endometria and ectopic lesions of endometriosis patients, whereas normal endometrial and nondiseased peritoneal tissues lacked detectable aromatase. Malik et al. (2018) performed immunohistochemical analysis of menstrual blood samples from endometriosis patients and nonendometriosis patients. They reported significantly increased P450 aromatase (CYP19A1) expression in endometriosis patients: 32.4% of the endometriosis patients presented moderate expression, and 67.6% presented strong expression, with no cases of negative or weak expression observed. These findings suggest that elevated aromatase levels in menstrual blood may serve as a potential noninvasive biomarker for endometriosis, offering insights for early diagnosis and disease management.

Ji et al. (2023) utilized data-independent acquisition coupled with mass spectrometry and bioinformatics to quantify differentially expressed proteins in menstrual blood. They reported significantly upregulated expression of Chemokine Ligand 5 and Interleukin-1 Receptor Antagonist in endometriosis patients. These findings highlight Chemokine Ligand 5 and Interleukin-1 Receptor Antagonist as promising candidates for endometriosis diagnosis, advancing biomarker research in this field.

### 2.3 Genital tuberculosis

Genital tuberculosis typically arises as a complication of pulmonary or extrapulmonary tuberculosis and spreads via blood or lymphatic pathways (Aliyu et al., 2004). Female genital tuberculosis (FGTB) is a clinically silent chronic disease that primarily involves the fallopian tubes in nearly all cases, leading to infertility, dyspareunia, menstrual irregularities, and chronic pelvic inflammation (Namavar et al., 2001). Owing to its atypical presentation, such as infertility or mild pelvic pain, which often overlaps with symptoms of other gynecological conditions, such as pelvic inflammatory disease or endometriosis, diagnosis requires the integration of patient history, imaging (e.g., ultrasound or MRI), and mycobacterial testing (Bose, 2011). Currently, no single diagnostic test is sufficient for FGTB confirmation, necessitating a multidisciplinary approach.

Paine et al. (2018) demonstrated that MB analysis via multiplex polymerase chain reaction offers a noninvasive alternative for FGTB diagnosis. The method achieved 90.2% sensitivity and 86.1% specificity, significantly outperforming traditional endometrial-based approaches. By analyzing MB samples without requiring invasive procedures such as dilation and curettage (D&C) or laparoscopy and delivering results within hours, this approach addresses the critical limitations of conventional diagnostics. Clinically, rapid, noninvasive MB testing enables early FGTB detection, allowing timely antitubercular therapy initiation to improve fertility outcomes and quality of life. In resource-limited settings, it circumvents costly and painful procedures such as D&C, reducing healthcare costs and patient discomfort. Additionally, the high sensitivity and specificity of multiplex polymerase chain reaction provide a reliable screening tool for asymptomatic or early-stage FGTB, particularly in reproductive-aged women, minimizing diagnostic delays and misdiagnosis risks while advancing clinical practice.

## 3 Disease treatment

**FIGURE 1:**
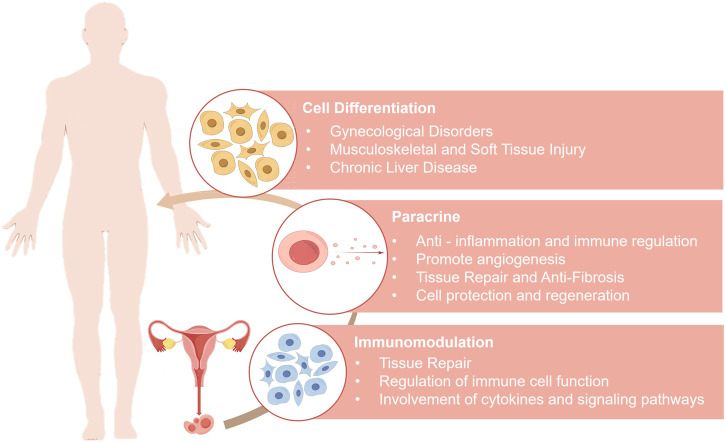
Mechanisms of menstrual blood-derived stem cells in disease treatment.

MenSCs hold significant promise in the field of regenerative medicine (Figure 1). Clinically, MenSCs exhibit low immunogenicity and can be expanded for more than 20 passages *in vitro*. This immune privilege is attributed to their low expression of major histocompatibility complex class II molecules (MHC-II, specifically HLA-DR) and the absence of co-stimulatory molecules (CD80/CD86), supporting their capacity for immune evasion (Khoury et al., 2014). Both preclinical and clinical studies have demonstrated that MenSC transplantation does not elicit immune rejection or severe adverse effects (Khanjani et al., 2014; Zhong et al., 2009; Bockeria et al., 2013).

In addition to MenSCs, other cell types derived from menstrual blood—such as endometrial regenerative cells (ERCs) and endometrial stromal cells (ESCs)—can also be easily obtained and have been widely applied in the research and treatment of various diseases. Overall, menstrual blood-derived cells represent a valuable source for stem cell-based therapies, with broad therapeutic potential across a range of medical conditions.

### 3.1 Therapeutic potential of cell differentiation

MenSCs harness their pluripotency and self-renewal capacity to differentiate into various functional cell types under specific *in vitro* induction conditions, thereby restoring or replacing damaged tissues and achieving tissue regeneration. Their differentiation potential spans multiple tissue and cell types, demonstrating notable therapeutic potential in the treatment of a variety of diseases.

#### 3.1.1 Treatment of gynecological disorders

##### 3.1.1.1 Infertility

For women with unexplained infertility, endometrial functional abnormalities may contribute to the underlying pathology. Cell-based therapies, particularly those that leverage the capacity of stem cells for decidualization or epithelial differentiation, offer a potential solution to improve endometrial function. Such therapies promote endometrial tissue remodeling, creating optimal conditions for embryo implantation. Piersma et al. (2015) successfully induced decidualization in MenSCs, observing the upregulation of characteristic decidualization markers such as prolactin, progesterone receptor, estrogen receptor, and insulin-like growth factor-binding protein. Additionally, key genes involved in decidualization including FOXO1, NOTCH1, NANOG, WNT4, KLF4, OCT4, SOX2, and LIN28A, as well as angiogenesis-related genes such as HIF1A, VEGFR-2, and VEGFR-3, were significantly upregulated. This coordinated gene expression enhances endometrial functionality, providing a novel direction for cellular replacement therapies in the treatment of infertility.

##### 3.1.1.2 Ovarian disorders

Premature ovarian insufficiency (POI) is characterized by menstrual irregularities (amenorrhea, oligomenorrhea, or polymenorrhea) in women under 40 years of age and is accompanied by elevated follicle-stimulating hormone (FSH) levels (>25 U/L) and fluctuating estrogen decline (Feng et al., 2019). Studies have demonstrated that the transplantation of MenSCs improves ovarian function in POI models through multiple mechanisms. When transplanted via the tail vein into cyclophosphamide-induced POI model mice, MenSCs regulate follicular development, restore estrous cyclicity, reduce ovarian apoptosis, and maintain microenvironmental homeostasis via ECM-dependent FAK/AKT signaling activation (Feng et al., 2019). MenSCs also significantly improve physiological parameters in mice with ovarian damage, including serum hormone levels (e.g., FSH and estradiol (E2)), body weight, estrous cyclicity, ovarian reserve markers (e.g., anti-Müllerian hormone and FSH receptor (FSHR)), and follicle counts (Lai et al., 2015; Liu et al., 2014). Gene expression analysis revealed that posttransplantation ovarian gene expression profiles in mice closely resemble those of human ovarian tissue, suggesting that the POI microenvironment may induce MenSCss differentiation into ovarian-like cells (Liu et al., 2014).

Furthermore, the ability of MenSCs to promote germ cell differentiation has increased their therapeutic utility in treating ovarian disorders. Lai et al. (2016) demonstrated that MenSCs exposed to follicular fluid *in vitro* differentiate into oocyte-like cells and theca-like cells expressing FSHR and luteinizing hormone receptor, with steroidogenic capacity. Under coculture conditions, MenSCs further generate multinucleated embryoid structures, indicating their potential for oocyte development.

##### 3.1.1.3 Intrauterine adhesions

Intrauterine adhesion (IUA), an acquired disorder following endometrial injury, is characterized by inadequate endometrial thickness, resulting in infertility and pregnancy loss even after assisted reproduction. Malik et al. (2018) demonstrated that MenSCs differentiated toward the endometrial lineage via PDGF, TGF-β, EGF, and 17β-estradiol expressed endometrial markers (cytokeratin (CK), vimentin, estrogen receptor, and progesterone receptor (PR)) in NOD-SCID mice posttransplantation, suggesting their capacity to reconstruct the endometrial architecture and restore fertility.

Fibrosis in IUA involves TGF-β-induced myofibroblast differentiation of endometrial stromal cells and the upregulation of α-smooth muscle actin (αSMA), type I collagen, CTGF, and fibronectin while impairing ESC migration (Deans and Abbott, 2010; Piersma et al., 2015; Zhu et al., 2019). Zhu et al. (Deans and Abbott, 2010) revealed that MenSCs-endometrial stromal cells coculture suppressed TGF-β-driven fibrosis by activating Hippo signaling (via TAZ phosphorylation), restoring ESC migratory capacity and promoting endometrial repair.

Clinical research has further verified the therapeutic potential of MenSCs. MenSCs isolated from the menstrual effluent of seven severe IUA patients were injected into the uterine cavity with hormone therapy, resulting in increased endometrial thickness in all patients (Malik et al., 2018). Among these, three achieved pregnancy with one live birth, demonstrating the efficacy of MenSCs in improving endometrial function and fertility outcomes (Malik et al., 2018; Chen et al., 2016).

#### 3.1.2 Treatment of musculoskeletal and soft tissue injury

MenSCs exhibit remarkable potential in the treatment of musculoskeletal disorders and the repair of soft tissue injuries. Through directed differentiation, these cells can be induced to transform into osteoblasts, chondrocytes, and adipocytes, offering new therapeutic avenues for the repair of bone injuries, degenerative diseases, and congenital defects.

In the context of bone and cartilage regeneration, MenSCs cultured in osteogenic induction medium for 21 days presented marked morphological changes, along with significantly upregulated expression of key osteogenic markers such as alkaline phosphatase (ALP), secreted phosphoprotein 1 (SPP1), and bone gamma-carboxyglutamic acid-containing protein (BGLAP) (Skliutė et al., 2021). During chondrogenic differentiation, increased expression of the collagen type II alpha 1 (COL2A1) gene further supports its regenerative potential in skeletal and cartilage tissues.

With respect to muscle regeneration and pelvic organ prolapse (POP), the ability of MenSCs to differentiate into smooth muscle cells provides new insights for POP repair. The pathological basis of POP involves degeneration of vaginal smooth muscle, leading to loss of pelvic floor support (Chen et al., 2016). In a study by Chen et al. (2016), MenSCs were successfully induced to differentiate into smooth muscle cells under TGF-β1 stimulation via the TGFBR2/ALK5/Smad2/3 signaling pathway, laying the groundwork for cell-based therapies. Furthermore, in a clinical study of Duchenne muscular dystrophy (DMD), a patient who received an intramuscular injection of 116 million MenSCs presented significantly improved muscle strength and restored dystrophin expression (Ichim et al., 2010), highlighting the therapeutic potential of MenSCs in muscle repair.

In the context of soft tissue regeneration, the adipogenic potential of MenSCs is critical. Under rosiglitazone induction, the mRNA expression of adipogenic markers—including leptin receptor (LEPR), peroxisome proliferator-activated receptor gamma (PPAR-γ), and lipoprotein lipase (LPL)—was significantly upregulated (Khanmohammadi et al., 2014). Compared with BM-MSCs, MenSCs offer advantages in terms of accessibility and ethical acceptability, making them ideal candidates for repairing soft tissue defects resulting from burns or tumor resection.

The application of MenSCs also extends to cutaneous wound healing. These cells can differentiate into mature keratinocytes and express epidermal-specific markers *in vitro*, including keratin 14 (K14), p63, and involucrin (IVL) (Fard et al., 2018; Akhavan-Tavakoli et al., 2017). This epithelial differentiation capacity positions MenSCs as promising cell sources for the treatment of skin injuries such as burns and ulcers.

#### 3.1.3 Treatment of chronic liver disease

MenSCs demonstrate significant potential for hepatocyte differentiation in the treatment of chronic liver diseases, suggesting a novel direction for regenerative medicine. Studies indicate that under the induction of factors such as hepatocyte growth factor (HGF) and Oncostatin M (OSM), MenSCs differentiate into hepatocyte-like cells, with the degree of differentiation showing a positive correlation with the concentrations of these factors (Malik et al., 2018). Differentiated cells express key hepatocyte markers—including albumin, tyrosine aminotransferase (TAT), and cytokeratin-18 (CK18)—at both the mRNA and protein levels and exhibit essential hepatocyte functions, such as albumin secretion, glycogen storage, and cytochrome P450 7A1 expression, thereby effectively mimicking the metabolic and detoxification functions of mature hepatocytes (Malik et al., 2018). Additionally, induced ERCs have been shown to differentiate into functional hepatocyte-like cells (Khademi et al., 2014). These findings suggest that menstrual blood-derived cells offer promising new avenues for cell-based therapies in chronic liver diseases, particularly in the regenerative treatment of hepatic fibrosis and cirrhosis.

#### 3.1.4 Expanded therapeutic potential of MenSCs through multilineage differentiation

Multilineage-differentiating stress-enduring (Muse) cells, derived from mesenchymal stem cells (MSCs), are pluripotent cells with the capacity to differentiate into all three germ layers and exhibit enhanced resistance to environmental stress (Heneidi et al., 2013). Muse cells can evade immunological barriers, such as the pulmonary capillary network (Li et al., 2013a), and preferentially home to sites of tissue injury (Kushida et al., 2018), thereby overcoming the limitations associated with conventional MSCs in clinical applications. Li et al. (2024) successfully isolated Muse cells from MenSCs via prolonged trypsin digestion and demonstrated their significant therapeutic efficacy in animal models of acute liver injury and intracerebral hemorrhage, with superior homing ability and treatment outcomes compared with those of conventional MSCs.

Induced pluripotent stem cells (iPSCs) are pluripotent cells reprogrammed from somatic cells without relying on embryonic sources (Li et al., 2013b). iPSCs exhibit gene expression, pluripotency, and epigenetic profiles highly similar to those of embryonic stem cells (Park et al., 2008a; Ratajczak et al., 2008; Ye et al., 2009; Lengerke and Daley, 2010), providing an ethically acceptable cell resource for disease modeling, drug development, and regenerative medicine. Although iPSCs can be generated from various somatic cell sources (Takahashi et al., 2007; Yu et al., 2007; Hockemeyer et al., 2008; Huangfu et al., 2008; Lowry et al., 2008; Park et al., 2008b; Aasen et al., 2008; Kim et al., 2009; Sun et al., 2009; Li et al., 2010; Zhao et al., 2010; Zhou et al., 2011), traditional sources such as human dermal fibroblasts (HDFs) require invasive skin biopsies and prolonged *in vitro* expansion, limiting their practical utility. In contrast, MenSCs, owing to their noninvasive sourcing, ease of acquisition, robust proliferation, and stem cell-like phenotype (Malik et al., 2018; Patel et al., 2008), represent ideal candidates for iPSC generation.

### 3.2 Therapeutic potential via paracrine mechanisms

The therapeutic effects of MSCs are primarily mediated through their paracrine activity, which involves the secretion of bioactive factors—such as growth factors, chemokines, cytokines, and EVs—to modulate the local microenvironment (Pittenger et al., 2019; Fan et al., 2020; Wang et al., 2015; Seo et al., 2021; Liang et al., 2014; Chang et al., 2021; Razavi et al., 2020). With increasing research on MenSCs, the therapeutic potential of their paracrine mechanisms has been validated in various diseases. Factors secreted by MenSCs can modulate immune responses, promote tissue regeneration, inhibit inflammation and fibrosis, and enhance angiogenesis, thereby offering novel therapeutic strategies for chronic disease management and regenerative medicine.

#### 3.2.1 Treatment of gynecological diseases

In the treatment of gynecological diseases, the paracrine activity of MenSCs has multiple therapeutic potential. The low success rate of *in vitro* fertilization (IVF) in older women is closely associated with abnormal reactive oxygen species (ROS) metabolism in oocytes (Mihalas et al., 2017). Studies have shown that EVs secreted by MenSCs can function as exogenous ROS scavengers, thereby reducing the dependence of embryos from aged female mice on endogenous antioxidant proteins such as superoxide dismutase 1 (SOD1) and glutathione peroxidase 1 (GPX1), ultimately improving IVF outcomes (Marinaro et al., 2018). Proteomic analyses further suggest that gene expression changes related to oxidative stress (GPX1, superoxide dismutase 1), metabolism (ACACA, GAPDH), placentation (PGF, VEGF-A), and stem cell differentiation (POU5F1, SOX2) may underlie the observed improvement in embryo quality (Marinaro et al., 2018; Marinaro et al., 2019).

In intrauterine adhesion (IUA) repair, MenSCs transplantation has been shown to accelerate endometrial regeneration. Zhang et al. (2016) reported that after being transplanted into a mouse model of endometrial injury, MenSCs activated the AKT and MAPK signaling pathways and upregulated angiogenesis-related proteins such as eNOS, VEGFA, VEGFR1, VEGFR2, and Tie2. These effects significantly shortened the repair time (complete regeneration within 7 days) and improved pregnancy rates and fetal counts (Huang et al., 2015; Lv et al., 2016). Additionally, fibroblast growth factor 2 secreted by MenSCs further promotes angiogenesis and cellular proliferation, thereby attenuating ovarian fibrosis and restoring sex hormone levels (Seo et al., 2013).

In the treatment of poor ovarian response (POR), paracrine factors from MenSCs modulate the local microenvironment to promote follicular development, as well as the differentiation of embryonic-like and ovarian stem cells. This leads to significant improvements in oocyte yield and quality, fertilization rates, embryo development rates, pregnancy rates, and live birth rates (Zafardoust et al., 2020; Rajabi et al., 2018; Bhartiya, 2018).

Moreover, the paracrine effects of MenSCs have shown their ability to inhibit epithelial ovarian cancer (EOC). Bu et al. (2016) demonstrated that MenSCs increased the proapoptotic Bax/Bcl-2 and Bad/Bcl-xL ratios, reduced the mitochondrial membrane potential, and activated the intrinsic apoptotic pathway to suppress EOC cell proliferation. Furthermore, they inhibited EOC cell cycle progression by blocking AKT phosphorylation. These findings suggest a promising strategy for the use of MenSCs in EOC therapy.

#### 3.2.2 Treatment of liver diseases

The paracrine effects of MenSCs have significant potential in the treatment of liver diseases. In nonalcoholic fatty liver disease, the progression of which is closely associated with metabolic dysregulation—such as insulin resistance and lipid accumulation (Zhao et al., 2017)—MenSCs have been shown to modulate the expression of the key gene Rnf186 by secreting HGF. Rnf186 interferes with insulin signaling and lipid synthesis, and its abnormal expression is suppressed by the paracrine action of MenSCs, thereby alleviating metabolic disturbances (Du et al., 2023).

In models of cholestatic liver injury, MenSCs repair tissue damage via paracrine mechanisms. Yang et al. (2022) demonstrated that MenSCs significantly enhanced survival in mice while reducing the serum levels of liver injury markers (AST, ALT, ALP, DBIL). This effect is attributed to the upregulation of hepatic β-catenin, which helps to restore the integrity of tight junctions and normalize the expression of bile transport proteins (OATP2, BSEP, and NTCP1), ultimately inhibiting intrahepatic bile duct dilation, cholestasis, and the progression of fibrosis.

With respect to liver fibrosis, MenSCs secrete an array of factors that collectively form a synergistic paracrine network. For example, monocyte chemoattractant protein-1 recruits immune cells to the injured area for targeted inflammation regulation (Chen et al., 2017a), whereas Interleukin (IL)-6 and IL-8 stimulate proliferative pathways in hepatic cells and promote the recruitment of neutrophils and macrophages, thereby enhancing tissue repair (Qiu et al., 2012; Cai et al., 2008). Moreover, HGF secreted by MenSCs inhibits the activation of hepatic stellate cells (e.g., the LX-2 cell line), directly impeding fibrogenesis (Chen et al., 2017a). In addition, angiopoietin stimulates cell proliferation and survival (Li and Hu, 2012) and, together with Axl, coregulates apoptotic and proliferative signaling (D’Ar et al., 2002). IGFBP-6 prevents fibroblast apoptosis (Qiu et al., 2012; Micutkova et al., 2011), further mitigating the chronic inflammation that drives fibrosis. Collectively, these paracrine factors contribute to a therapeutic network that counteracts the progression of liver fibrosis.

For hepatocellular carcinoma (HCC) treatment, MenSCs exert their therapeutic effects through epigenetic modulation. In a time-dependent manner, their paracrine secretions can restore the levels of 5-hydroxymethylcytosine (5-hmC) and TET1 in HCC cells (Wu et al., 2019). TET1 and TET2 regulate DNA demethylation to suppress aberrant demethylation in HCC cells (Chen Q. et al., 2017; Ko et al., 2010). Upon activation by MenSCs, increased enrichment of 5-hmC and 5-mC in key enhancer regions leads to downregulation of the PI3K/AKT and RAF/ERK pathways, thereby inhibiting HCC cell proliferation and promoting apoptosis. Moreover, MenSCs modulate the DNA modification of chemoresistance-related genes, such as ID4 and HMGA1, enhancing their therapeutic efficacy against HCC(96). These findings suggest that the paracrine mechanisms of MenSCs, which involve the targeting of oncogenic gene expression through epigenetic regulation, constitute a novel therapeutic strategy for HCC.

#### 3.2.3 Treatment of pulmonary diseases

Idiopathic pulmonary fibrosis is a chronic and progressive interstitial lung disease characterized by excessive fibrosis of the lung tissue and is associated with high morbidity and mortality rates (Blackwell et al., 2014). Although lung transplantation remains the most effective treatment, its clinical application is limited by donor scarcity, and existing pharmacological interventions provide only modest antifibrotic efficacy. Chen et al. (2020a) demonstrated that MenSCs transplantation alleviated fibrosis in bleomycin-induced Idiopathic pulmonary fibrosis models by homing to injured sites, suppressing epithelial cell apoptosis and proinflammatory cytokine (e.g., IL-1β and TNF-α) secretion, and inhibiting myofibroblast activation and collagen deposition.

ERCs also exhibit multipotent therapeutic potential in Idiopathic pulmonary fibrosis treatment (Zhao et al., 2018a). On the one hand, ERCs suppress TGF-β signaling and downregulate the proinflammatory cytokines IL-1β and TNF-α by upregulating IL-10 (Sziksz et al., 2015). They also restore total superoxide dismutase (T-SOD) activity to reduce oxidative stress and modulate apoptotic signaling via downregulation of the Bax/Bcl-2 ratio, contributing to alveolar epithelial cell protection. On the other hand, ERCs upregulate antifibrotic genes such as HGF and matrix metalloproteinase-9 (MMP-9), further inhibiting fibrosis progression.

In acute lung injury animal models, MenSCs also exhibit therapeutic efficacy (Ren et al., 2018; Xiang et al., 2017). Through paracrine signaling, MenSCs increase proliferating cell nuclear antigen (PCNA) expression to promote cell proliferation, inhibit caspase-3-mediated apoptosis to reduce cell death, increase the secretion of anti-inflammatory factors such as IL-10 and keratinocyte growth factor (KGF), and simultaneously suppress proinflammatory cytokines such as IL-1β, neutrophil infiltration, and myeloperoxidase (MPO) activity, thereby attenuating pulmonary inflammation and restoring lung function.

#### 3.2.4 Treatment of diabetic skin wounds

Under diabetic conditions, wound healing is disrupted through multiple mechanisms, including persistent inflammation, hypoxia, cellular dysfunction, impaired angiogenesis, and neuropathy (Guo and Dipietro, 2010). The chronic nature of diabetic wounds is closely associated with dysregulated inflammatory phase transitions. While inflammation is essential for initiating tissue repair (Aller et al., 2004), excessive activation of proinflammatory macrophages (M1) and the resulting prolonged inflammatory state significantly impede healing (Landén et al., 2016). The phenotypic switch from M1 to reparative macrophages (M2) is critical for tissue regeneration (Finley et al., 2016; Zhang et al., 2010; Emin et al., 2007; Murray et al., 2014); however, in the diabetic microenvironment, downregulation of M2-associated genes (e.g., Arg-1) leads to impaired polarization (Finley et al., 2016; Schwab et al., 2007). MenSC-Exos promote M2 polarization by modulating inducible nitric oxide synthase activity and the ARG: inducible nitric oxide synthase ratio, with a sustained increase in the M2/M1 ratio observed between days 7–14 posttreatment (Dalirfardouei et al., 2019; Mirzadegan et al., 2022).

Macrophage function is also closely linked to neural regeneration. As the primary source of neuroprotectin D1 (NPD1), M2 macrophages may facilitate axonal regeneration and contribute to wound repair (C et al., 2013; Hong et al., 2014). Mirzadegan et al. (Mirzadegan et al., 2022) demonstrated that MenSC intervention significantly increased the density of protein-gene product 9.5 (PGP9.5)-positive nerve fibers in diabetic wounds, suggesting the potential for reversing cutaneous neuropathy. At the molecular level, MenSC-Exos activate the NF-κB signaling pathway to increase keratinocyte proliferation and differentiation (Dalirfardouei et al., 2019; Brantley et al., 2001). Additionally, through interactions with the Notch pathway (Na et al., 2017; Shi et al., 2015) and Jumonji domain-containing protein D3, they may regulate epigenetic remodeling in keratinocytes (Na et al., 2016), thereby promoting migration and re-epithelialization.

The balance of collagen metabolism is crucial for optimal wound healing. MenSC-Exos induce the expression of type I collagen and type III collagen mRNAs, initially promoting type III collagen synthesis to support granulation tissue formation (Volk et al., 2011). At later stages, they reduce the type I collagen/type III collagen ratio to suppress scar hyperplasia (Yates et al., 2012; Cuttle et al., 2005; Larson et al., 2010; Zhang et al., 2015).

In terms of angiogenesis, MenSC-Exos dose-dependently upregulated key angiogenic markers, including vascular endothelial growth factor A (VEGFA), CD31, and von Willebrand factor (vWF), with 10 μg of MenSC-Exos demonstrating tenfold greater efficacy than exosomes from other stem cell sources (Dalirfardouei et al., 2019; Mirzadegan et al., 2022; Hu et al., 2016; Liang et al., 2016).

Importantly, although MenSCs exhibit strong migratory capacity due to high CXCR4 expression (Luz-Crawford et al., 2016), dysfunctional fibroblasts in diabetic wounds often fail to produce stromal cell-derived factor-1, thereby compromising MenSC homing efficiency (Mirzadegan et al., 2022; Brem et al., 2007). This paradox underscores the critical role of the local microenvironment in modulating stem cell-based therapies.

#### 3.2.5 Treatment of central nervous system disorders

Spinal cord injury (SCI), characterized by sensory and motor dysfunction, continues to pose major clinical challenges because of the limited therapeutic efficacy of current treatment paradigms (Kunte et al., 2015). Recent studies have demonstrated that MenSCs transplantation promotes neural repair via multiple mechanisms. Wu et al. (2018) confirmed that MenSCs increase the expression of brain-derived neurotrophic factor, thereby supporting neuronal survival and axonal regeneration (Uchida et al., 2016). Additionally, MenSCs upregulate mature neuronal markers such as neurofilament-200 (NF-200) and microtubule-associated protein-2 (MAP-2), facilitating structural reconstruction of neural tissue (Li et al., 2013a). MenSCs also contribute to neural repair by attenuating glial scar formation, as evidenced by the reduced expression of inhibitory molecules such as chondroitin sulfate proteoglycans (CSPGs) (Anderson et al., 2016; Fan et al., 2017). Concurrently, MenSCs suppress the production of proinflammatory cytokines, including TNF-α and IL-1β, thereby alleviating neuroinflammation. In animal models, local injection of MenSCs significantly improved hindlimb motor function in rats with SCI(131).

Hjazi and colleagues (Hjazi et al., 2024) further advanced this therapeutic approach by combining MenSC-Exos with hyperbaric oxygen therapy (HBOT) in a model of traumatic SCI (TSCI). While MenSC-Exos exert anti-inflammatory and antiapoptotic effects (Dalirfardouei et al., 2019), HBOT reduces neuronal apoptosis by downregulating the expression of proinflammatory cytokines (e.g., IL-1β and TNF-α) (Ghaemi et al., 2023; Zhao et al., 2021). The combined therapy synergistically regulated apoptosis, oxidative stress, and inflammation, resulting in improved histopathological outcomes and functional recovery.

Alzheimer’s disease, the most common type of dementia, is pathologically defined by amyloid-beta (Aβ) plaques and neurofibrillary tangles (NFTs). Aβ originates from proteolytic cleavage of the transmembrane amyloid precursor protein (APP), whereas NFTs consist of hyperphosphorylated and misfolded tau proteins (Ballard et al., 2011). Zhao et al. (2018b) reported that MenSCs transplantation reduced Aβ deposition in the hippocampus and cortex, potentially by regulating aberrant APP processing, enhancing Aβ clearance by microglia, and suppressing tau hyperphosphorylation. Moreover, MenSCs promoted the transformation of microglia into a neuroprotective phenotype, facilitating anti-inflammatory cytokine secretion and markedly improving cognitive deficits in APP/PS1 transgenic mice.

Notably, Lopez-Verrilli et al. (2016) reported a biphasic effect of MenSCs on neurite outgrowth. While direct contact between MenSCs and neurons inhibited neurite elongation, their secreted factors—including VEGF, HGF, and brain-derived neurotrophic factor—promoted axonal extension. Further investigation revealed that the exosomal fractions of MenSC-EVs promoted neurite outgrowth, whereas the microvesicle fractions had inhibitory effects. This functional heterogeneity provides new insights into the application of MenSCs in treating neurodegenerative diseases.

#### 3.2.6 Treatment of other systemic diseases

Myocardial infarction (MI), an acute cardiovascular emergency, is primarily treated by curtailing the progression of myocardial necrosis and promoting functional recovery (Lu et al., 2015). Wang et al. (2017) reported that extracellular vesicles derived from MenSC-EVs regulate the PTEN/Akt pathway through high expression of miR-21. This mechanism both inhibits cardiomyocyte apoptosis and promotes angiogenesis, thereby significantly improving cardiac function in infarcted rat models. This discovery provides a novel strategy for myocardial regeneration therapy.

In the field of prostate cancer (PC) treatment, Alcayaga-Miranda et al. (2016) revealed that MenSC-Exos reduce tumor cell ROS levels and downregulate the expression of vascular endothelial growth factor (VEGF) and hypoxia-inducible factor-1α (HIF-1α). This disrupts the tumor’s hypoxic adaptation, thereby inhibiting tumor hemoglobin biosynthesis and neovascularization, which contributes to an antitumor effect. Such microenvironmental modulation offers a new avenue for targeted therapy in solid tumors.

With respect to ulcerative colitis (UC), (Malik et al., 2018) demonstrated that ERCs exert anti-inflammatory effects by rebalancing cytokine profiles—reducing IL-2 and TNF-α while increasing IL-4 and IL-10 levels. Further research has shown that preconditioning with stromal cell-derived factor-1 enhances CXCR4 expression in ERCs, promoting M2 macrophage polarization and Treg cell generation (Li et al., 2019); prestimulation with IL-1β inhibits the DKK1 protein and activates the Wnt/β-catenin pathway, thereby strengthening their immunomodulatory function (Yu et al., 2021); and melatonin preconditioning enhances ERC efficacy via antioxidant protection, significantly lowering both the disease activity index and tissue damage scores (Hao et al., 2024).

In the treatment of type 1 diabetes mellitus (T1DM), (Malik et al., 2018) confirmed that intravenous infusion of MenSCs results in targeted migration to the injured pancreas. This migration activates Ngn3-positive endocrine progenitor cells, facilitating β-cell regeneration, effectively reversing hyperglycemia, increasing insulin secretion, and restoring islet architecture. This finding provides a potential solution to the shortage of islet donors.

In sepsis treatment research, (Alcayaga-Miranda et al., 2015a) reported that MenSCs enhance host defense by increasing the expression of antimicrobial peptides and hepcidin. When combined with antibiotics, this approach significantly improves bacterial clearance and survival. Notably, MenSC-Exos exert a protective effect against LPS-induced liver injury by modulating abnormal natural killer (NK) cell activation (Chen et al., 2017c), thereby reducing inflammatory organ damage and demonstrating multitarget therapeutic potential.

#### 3.2.7 Molecular mediators and signal transduction cascades

MenSCs exert reparative effects via the paracrine release of small extracellular vesicles (sEVs), which mediate multiple signaling pathways. miRNA profiling of exosomes has identified the enrichment of key microRNAs (miRNAs), such as miR-21 and miR-lethal-7 (let-7), in MenSC-derived sEVs. miR-21 enhances cell survival and improves cardiac function by targeting PTEN and activating the AKT/PKB signaling cascade (Wang et al., 2017). Let-7 modulates the NLRP3 pathway, enhancing the inhibition of ROS production and mitochondrial DNA damage via suppression of lectin-like oxidized low-density lipoprotein receptor-1 (LOX-1), thereby facilitating the repair of alveolar epithelial cells (Sun et al., 2019). In pancreatic regeneration, sEVs activate the PDX-1 signaling axis to promote β-cell regeneration and insulin secretion (Mahdipour et al., 2019).

In terms of immune regulation, MenSC-Exo induce phosphorylation of STAT3/STAT6, driving M2 macrophage polarization (Liu et al., 2017). Similarly, CD73 present in exosomes from endometrial regenerative cells promotes M2 polarization through a MAPK-dependent hydrolysis of ATP, thereby contributing to renal protection (Shao et al., 2025).

Regarding metabolic regulation, hepatocyte growth factor (HGF) secreted by MenSCs downregulates hepatic Rnf186 expression and modulates the AMPK-mTOR signaling pathway, improving glucose and lipid metabolism. In addition, HGF upregulates β-catenin, contributing to the repair of tight junctions and the expression of bile transporters (OATP2, BSEP, NTCP1), thereby attenuating the progression of hepatic fibrosis (Du et al., 2023; Yang et al., 2022).

At the epigenetic level, MenSCs restore the expression of TET1/2 and 5-hmC levels in hepatocellular carcinoma cells in a time-dependent manner. By modulating the 5-hmC/5-methylcytosine enrichment at enhancer regions of PI3K/AKT and RAF/ERK pathway target genes, MenSCs relieve repression of FOXO3, subsequently promoting downstream apoptotic processes (Wu et al., 2019).

### 3.3 Therapeutic effects mediated by immunomodulation

MenSCs exhibit unique advantages in the field of immunotherapy. Like BM-MSCs, MenSCs express low levels of HLA-B and HLA-C and lack HLA-DR expression, indicating potential immune privilege properties (Luz-Crawford et al., 2016). However, further investigations revealed distinct immunoregulatory features compared with those of BM-MSCs. Notably, MenSCs express lower levels of the Toll-like receptors TLR3 and TLR4 and exhibit weaker suppression of T cell subsets under low-concentration conditions (Luz-Crawford et al., 2016). In specific microenvironments, they may even stimulate lymphocyte proliferation (Nikoo et al., 2012). These distinctive immunomodulatory properties offer an important theoretical basis for the development of novel immunotherapeutic strategies.

#### 3.3.1 Effects on tissue repair

ERCs demonstrate multifaceted immunomodulatory capabilities during tissue repair. Studies have shown that ERCs can induce the polarization of M2 macrophages, tolerogenic dendritic cells, and regulatory T and B cells both *in vivo* and *in vitro*. They also significantly suppress IgG and IgM antibody deposition and infiltration of CD4^+^ and CD8^+^ T cells. These immunoregulatory effects are closely associated with stromal cell-derived factor-1–mediated immunosuppressive mechanisms (Xu et al., 2017).

#### 3.3.2 Regulation of immune cell function

MenSCs exert notable regulatory effects on various immune cell populations. In reproductive immunology, uterine NK cells play essential roles in early pregnancy tolerance through killer cell immunoglobulin-like receptors (Male and Moffett, 2023). Shokri et al. reported that IFN-γ- and IL-1β-pretreated MenSCs inhibited NK cell cytotoxicity via the IL-6 and TGF-β pathways, thereby reducing perforin and granzyme production and promoting an immune-tolerant microenvironment conducive to pregnancy (de Pedro et al., 2021). Additionally, Hosseini et al. reported a significant decrease in TCRγδ cell proportions in the menstrual blood of patients with recurrent spontaneous abortion, suggesting a possible link to pregnancy-related immune dysregulation (Hosseini et al., 2016). With respect to dendritic cells, Bozorgmehr and colleagues demonstrated that MenSCs dose-dependently suppress monocyte differentiation into immature dendritic cells, possibly through high secretion levels of IL-6 and IL-10 (Bozorgmehr et al., 2014). In antitumor immunity, menstrual blood-derived immune cells also show substantial potential. Qin et al. isolated DC-CIK cells from the menstrual blood of ovarian cancer patients and reported that these cells inhibited ovarian cancer stem cells by activating the TNFR1–ASK1–AIP1–JNK signaling pathway (Qin et al., 2018).

#### 3.3.3 Involvement of cytokines and signaling pathways

Although MenSCs lack HLA-DR expression, limiting their antigen-presenting capacity, they exert immunoregulatory effects through the secretion of various paracrine molecules, including IL-6, TGF-β, COX-2, PGE2, indoleamine 2,3-dioxygenase, and programmed death ligand-1 (Malik et al., 2018; Luz-Crawford et al., 2016; Cuenca et al., 2018). Proteomic analysis revealed high expression levels of adhesion molecules such as activated leukocyte cell adhesion molecule and intercellular adhesion molecule-1 in MenSCs (Liu et al., 2018). These molecules interact with receptors such as LFA-1, directly participating in immune cell activation and regulation, and play critical roles in the immunomodulatory functions of MenSCs. Notably, MenSCs also secrete multiple angiogenic factors, including VEGF, HGF, angiogenin, and MMP-1, which contribute to both tissue regeneration and immune regulation.

## 4 Investigation of disease pathogenesis and therapeutic mechanisms

The pathogenesis of endometriosis remains incompletely understood; however, analysis of MenSCs from affected individuals has provided novel insights into its underlying mechanisms. Transcriptomic studies have shown significantly elevated levels of miR-200b-3p in MenSCs from patients with endometriosis (de Oliveira et al., 2022). This microRNA, which is commonly associated with tumor progression, regulates key cellular processes such as motility, proliferation, migration, and differentiation (Humphries and Yang, 2015). By suppressing the transcription factor ZEB1, miR-200b-3p reverses epithelial–mesenchymal transition, induces mesenchymal–epithelial transition, and modulates angiogenesis by targeting VEGF and epidermal growth factor receptor 2 (Wellner et al., 2009; Yang et al., 2016). These mechanisms may contribute to the proliferation, maintenance of stemness, and mesenchymal–epithelial transition of endometrial cells transported by retrograde menstruation, thereby promoting lesion formation and persistence.

The application of high-throughput technologies has further advanced our understanding of the molecular pathophysiology of endometriosis (Meola et al., 2010). However, these approaches require stable reference genes for accurate data interpretation. Zucherato et al. (2021) identified EIF2B1 and POP4 as the most stable reference genes in MenSCs derived from menstrual blood, whereas GAPDH and ACTB were found to be less reliable, providing an essential foundation for future experimental designs. Moreover, MenSCs from patients presented higher expression levels of the surface markers CD9, CD10, and CD29 than those from healthy individuals did, along with increased proliferative and invasive capacities (Nikoo et al., 2014). When cocultured with PB mononuclear cells, these MenSCs presented increased expression of indoleamine 2,3-dioxygenase-1 and COX-2, as well as elevated secretion of proinflammatory cytokines such as IFN-γ, IL-10, and monocyte chemoattractant protein-1, suggesting that MenSCs may contribute to disease progression through immunomodulation and altered cellular behavior (Nikoo et al., 2014).

High mobility group box-1 (HMGB1) also plays a critical role in endometriosis. HMGB1, a nonhistone nuclear protein abundant in mammalian chromatin, is involved in DNA transcription, repair, replication, and remodeling (Liu et al., 2010). Elevated HMGB1 levels have been detected in the blood of patients, where it interacts with receptors such as RAGE and TLR4, forming complexes with IL-1β or LPS to activate proinflammatory signaling pathways (Shimizu et al., 2017). Additionally, HMGB1 promotes endothelial cell proliferation and the release of angiogenic factors such as VEGF, thereby facilitating the implantation and survival of ectopic endometrial cells (Shimizu et al., 2017). Integrative omics analyses by Penariol et al. (2022) identified key genes (e.g., ATF3, ID1, ID3, and FOSB) and proteins (e.g., MT2A and COL1A1) involved in disease development. The chronic inflammatory microenvironment may disrupt MenSCs function, leading to dysregulation of gene and protein expression and contributing to the progression of endometriosis (Penariol et al., 2022).

In therapeutic investigations, the natural flavonoid components of Citrus latifolia have been utilized for their anti-inflammatory properties to alleviate dysmenorrhea and menorrhagia. Robeldo et al. (2020) demonstrated that Citrus latifolia increases the production of prostaglandin F2α, which acts on prostaglandin receptors to reduce the menstrual volume and inhibits the release of proinflammatory cytokines such as TNF-α (Ekström et al., 1991). This mechanism effectively blocks inflammatory signaling and nociceptive sensitization. Compared with nonsteroidal anti-inflammatory drugs, this approach is particularly effective in patients with menstrual disorders associated with insufficient prostaglandin F2α secretion (Robeldo et al., 2020).

## 5 Comparison with other mesenchymal stem cells

Phenotypically, MenSCs share similar surface markers with other mesenchymal stem cells (MSCs), such as CD10, CD29, CD44, CD73, CD90, and CD105 (Cordeiro et al., 2022; Margiana et al., 2022), and possess multilineage differentiation potential. However, MenSCs display a significantly faster proliferation rate, with a population doubling time of approximately 19.4 hours—roughly twice as fast as that of bone marrow-derived MSCs (BM-MSCs) (Skliutė et al., 2021). Functionally, their colony-forming unit-fibroblast frequency is 2–4 times higher than that of BM-MSCs, and their *in vitro* migratory ability is also superior.

In terms of paracrine activity, MenSCs secrete higher levels of VEGF and basic fibroblast growth factor, leading to enhanced angiogenesis. Notably, MenSCs induce hemoglobin production at levels 8-fold greater than BM-MSCs. They also exhibit superior feeder layer characteristics, supporting the expansion and maintenance of CD34^+^CD133+ hepatic stellate cells. MenSCs promote a 3-fold increase in cell expansion and generate more CFU-GEMM colonies (Alcayaga-Miranda et al., 2015b). By secreting growth factors and extracellular matrix proteins, MenSCs contribute to reconstructing the bone marrow microenvironment and support the *ex vivo* expansion of hematopoietic stem cells (Khoury et al., 2011).

Comparative studies in therapeutic applications also highlight MenSCs’ advantages (Wang et al., 2017). evaluated the effects of intramyocardial injection of BM-MSCs, adipose-derived MSCs, and MenSCs in a murine MI model. MenSCs exhibited superior cardioprotective effects and increased microvascular density, attributed to high miR-21 levels in their exosomes that activate the PTEN/Akt signaling pathway (Lopez-Verrilli et al., 2016). compared the effects of MenSCs, BM-MSCs, umbilical cord, and chorionic MSCs on cortical neurite outgrowth. MenSC-derived exosomes showed the strongest neurite-promoting effect, whereas their microvesicles inhibited neurite extension, suggesting functional specificity among extracellular vesicle subtypes. In liver regeneration, Fathi-Kazerooni et al. (Malik et al., 2018) demonstrated that MenSC-derived hepatocyte progenitor-like cells more effectively improved liver injury markers compared to BM-MSCs, with greater reductions in inflammation, necrosis, and collagen deposition. Mechanistically, this was linked to stronger anti-inflammatory effects exerted by MenSCs.

## 6 Comparison of menstrual blood and peripheral blood

In studies directly comparing MB and PB, (Iribarne-Durán et al., 2020) reported that MB samples contain substantial levels of endocrine-disrupting chemicals, notably parabens and benzophenones. These endocrine-disrupting chemicals concentrations are correlated with specific sociodemographic and lifestyle factors, such as the use of cosmetics, skin oils/creams, and hair dyes (Jiménez-Díaz et al., 2016). Importantly, MB levels of parabens and benzophenones were found to be lower than those measured in PB serum (Matsumoto et al., 2007), providing evidence for a blood–uterine barrier: a physical and/or metabolic partition that may restrict certain compounds from reaching the endometrium, potentially via glucuronidation-mediated metabolism and increased excretion of endocrine-disrupting chemicals. Further research revealed marked differences in immune cell composition between MB and PB. Hosseini et al. (2019) demonstrated that the frequencies of NKT cell subsets and their associated cytokine profiles differ significantly between MB and PB in fertile women, infertile women, and those with unexplained recurrent spontaneous abortion. This finding underscores the profound influence of the endometrial immune microenvironment on the cellular makeup of MB. Together, these studies not only validate MB as a promising biomarker for assessing endocrine-disrupting chemicals exposure and its link to gynecological conditions but also highlight its unique utility in probing local uterine immune mechanisms.

## 7 Current status of clinical trial research

Although preclinical studies in cellular and animal models have confirmed the safety and efficacy of MenSC transplantation, the clinical application of MenSCs remains limited compared to other stem cell sources. This is primarily due to the restricted availability of menstrual blood donors and the lack of standardized *in vitro* culture protocols. To date, clinical trials involving MenSCs have primarily evaluated their therapeutic effects in diseases such as multiple sclerosis, H7N9 influenza virus infection, severe COVID-19, Asherman’s syndrome, and ovarian insufficiency (Zhong et al., 2009; Zafardoust et al., 2020; Tan et al., 2016; Chen J. et al., 2020; Fathi-Kazerooni et al., 2022; Xu et al., 2021; Zafardoust et al., 2025; Zafardoust et al., 2023a; Zafardoust et al., 2023b). The results indicate that MenSC transplantation leads to marked clinical improvement in these conditions, with no serious adverse events or complications observed, underscoring the potential value of MenSCs in clinical applications.

In the context of ovarian insufficiency, the team led by Zafardoust has conducted long-term follow-up studies to assess the efficacy and potential complications of autologous MenSC therapy. Their clinical trial demonstrated that autologous MenSC transplantation can increase the rate of natural conception, improve ovarian function, and restore regular menstrual cycles. Importantly, no serious complications—such as endometriosis, ovarian malignancies, or autoimmune diseases—were reported, suggesting that autologous MenSC transplantation represents a safe and promising approach for improving reproductive outcomes in women with ovarian insufficiency (Table 1).

**TABLE 1:** Menscs-related clinical trials.

Subject condition	Subject number	Subject gender	Donor information	Follow-up time	Follow-up outcome	References
Multiple Sclerosis	4	Female	healthy, non-smoking, female volunteers between 18 and 30 years of age	2–12 months	no abnormalities	Zhong et al. (2009)
Asherman’s syndrome	7	Female	Autologous transplantation	3–6 months	recovery of endometrial morphology to a normal status	Tan et al. (2016)
Epidemic Influenza A (H7N9) Infection	17	Female	Healthy female donors (20–45 years old)	5 years	most clinical symptoms were ameliorative from 1 to 12 months	Chen et al. (2020b)
Severe COVID-19	14	Male and female	Master Cell Bank (derived from menses of at least five healthy women)	28 days	Reversal of hypoxia, immune reconstitution, and cytokine storm downregulation	Fathi-Kazerooni et al. (2022)
Severe and critically ill COVID‐19	28	Male and female (no statistical difference at the gender level)	3 healthy female donors (20–45 years old)	1 months	Significantly improved dyspnea	Xu et al. (2021)
Poor ovarian response	105	Female	Autologous MenSCs	3 years	Spontaneous pregnancy rate was significantly higher without complications	Zafardoust et al. (2025)
Poor ovarian response	15	Female	Autologous MenSCs	1 years	Spontaneous pregnancy rate was higher	Zafardoust et al. (2020)
Poor ovarian response	180	Female	Autologous MenSCs	2 months	Spontaneous pregnancy rate, anti-Mullerian hormone levels, and antral follicle count were significantly increased	Zafardoust et al. (2023a)
Premature ovarian failure	15	Female	Autologous MenSCs	1 years	Ovarian function improved and menses resumed	Zafardoust et al. (2023b)

However, current clinical research on MenSCs still faces limitations. Most studies involve small sample sizes and short follow-up durations, which weakens the statistical power for evaluating long-term safety despite supporting evidence from basic research. Therefore, large-scale, multicenter clinical trials are necessary to comprehensively assess the efficacy and safety of MenSC-based therapies. In addition, the majority of enrolled participants have been female, and only the study by Xu et al. (2021) has conducted sex-stratified analyses. Given the female-specific origin of MenSCs, sex-based stratification should be considered an essential component of future clinical investigations.

## 8 Conclusion

This review provides a comprehensive overview of the potential of MB in both basic research and clinical applications, with a particular emphasis on its innovative roles in disease diagnosis and therapy. As a biologically rich fluid containing diverse cellular and molecular constituents, MB offers a non-invasive, highly acceptable platform for investigating women’s reproductive health and pathology. In the therapeutic arena, MenSCs have emerged as a key resource in regenerative medicine owing to their pluripotency, low immunogenicity, and ease of procurement. Through mechanisms of direct differentiation, paracrine signaling, and immune modulation, MenSCs have demonstrated promising efficacy in preclinical models and early clinical studies.

## 9 Limitations

Nonetheless, the clinical translation of MenSC-based therapies faces several critical challenges—including *in vivo* cell tracking post transplantation, donor-related variability in therapeutic outcomes, the establishment of robust quality control and standardization protocols, and the need for long-term safety data. In addition, unresolved ethical and sociocultural considerations remain. Although the use of menstrual blood (MB) as a research sample raises fewer ethical concerns compared to embryonic or fetal tissues, ethical guidelines for MB collection are still evolving.

Moreover, cultural norms, religious beliefs, and disparities in education continue to pose barriers to MB donation, especially in low- and middle-income countries. In Islamic jurisprudence, menstruation is considered a state of ritual impurity, which may discourage donation. Similarly, in Hindu traditions, menstrual blood is sometimes perceived as carrying “negative energy,” potentially reducing donor willingness. In patriarchal societies, it is not uncommon for women to require spousal consent to donate biological materials, thereby limiting female autonomy. Trust may also be compromised when research teams are male-dominated, as some women may feel uncomfortable discussing menstrual health issues with male investigators.

## 10 Future directions

Despite these hurdles, ongoing technological and scientific advances are steadily unveiling the unique bioactive components and mechanisms of action of MB. To better preserve the three-dimensional architecture of the native extracellular matrix (ECM) and replicate the microenvironment required for cell proliferation and differentiation, scaffold-based technologies have been employed to facilitate the directed differentiation of MenSCs. Polylactic acid and multi-walled carbon nanotubes, as promising biomaterials, have been incorporated into scaffold designs due to their favorable mechanical properties and biocompatibility, enhancing MenSC differentiation toward germ cell lineages (Eyni et al., 2017).

A bilayer scaffold composed of amniotic membrane and silk fibroin has shown therapeutic potential in wound healing and skin regeneration. This scaffold mimics the skin ECM by providing optimized mechanical strength and 3D structure, significantly improving the regenerative capacity of MenSCs. On an immunomodulatory level, anti-inflammatory factors secreted by the amniotic membrane (e.g., SLPI/elafin) synergize with MenSC-derived paracrine cytokines such as IDO, PGE_2_, and TGF-β, promoting the polarization of macrophages from the pro-inflammatory M1 phenotype to the regenerative M2 phenotype. This scaffold system has also been shown to induce MenSC differentiation into keratinocyte-like cells (Mirzadegan et al., 2022), while upregulating VEGFA to activate CD34^+^ endothelial cells and stimulate angiogenesis. Concurrently, restoration of the SDF-1/CXCR4 signaling axis improves endothelial progenitor cell homing (Mirzadegan et al., 2020). *In vivo* studies in diabetic mouse models have demonstrated enhanced epidermal thickness and type I collagen synthesis following treatment, resulting in effective wound regeneration and healing (Araste et al., 2020).

Further translational progress has been made through large animal studies. For instance, (Emmerson et al., 2019) investigated the integration of endometrial MSCs with synthetic meshes in a sheep model of POP. Their findings showed that autologous eMSCs improved mesh biocompatibility and restored vaginal tissue strength, while exhibiting long-term survival *in vivo* and contributing to immune modulation, ECM remodeling, and fibrosis regulation.In parallel, organoid technologies are emerging as essential tools to study the menstrual cycle and endometrial diseases. Wiwatpanit et al. (2020) developed a scaffold-free endometrial organoid model consisting of epithelial and stromal components that closely mimics the physiology of native endometrium. This model provides a versatile 3D system to investigate hormone-induced pathological changes in the endometrium. Such insights are poised to drive the broader application of MB in regenerative and immune-based therapies, offering novel strategies to address persistent clinical needs.

Overall, MB represents a novel and promising tool for both disease diagnosis and therapeutic intervention. Its emerging applications in regenerative medicine and immunotherapy hold significant potential to address unresolved clinical challenges and advance the development of precision medicine.

## Abbreviations

MB, Menstrual Blood; MenSCs, Menstrual blood-derived stem cells; PB, Peripheral Blood; HPV, Human papillomavirus; CIN, Cervical intraepithelial neoplasia; NGS, Next-generation sequencing; TGF-β, Transforming growth factor-beta; BM-MSCs, Bone marrow-derived mesenchymal stem cells; POI, Premature ovarian insufficiency; FSH, Follicle-stimulating hormone; IUA, Intrauterine adhesion; VEGF, Vascular endothelial growth factor; HGF, Hepatocyte growth factor; EVs, Extracellular vesicles; ROS, Reactive oxygen species; IL, Interleukin; HCC, Hepatocellular carcinoma; MenSC-Exos, Menstrual blood-derived stem cell exosomes; Aβ, Amyloid-beta; NK, Natural killer; TNF-α, Tumor necrosis factor alpha; HMGB1, High mobility group box-1.
